# Midlife stress alters memory and mood-related behaviors in old age: Role of locally activated glucocorticoids

**DOI:** 10.1016/j.psyneuen.2017.12.018

**Published:** 2018-03

**Authors:** Nicola Wheelan, Christopher J. Kenyon, Anjanette P. Harris, Carolynn Cairns, Emad Al Dujaili, Jonathan R. Seckl, Joyce L.W. Yau

**Affiliations:** a Centre for Cardiovascular Science, University of Edinburgh, EH16 4TJ, United Kingdom; b Centre for Cognitive Aging and Cognitive Epidemiology, University of Edinburgh, EH8 8JZ, United Kingdom

**Keywords:** Glucocorticoids, Stress, 11β-HSD1, Anxiety, Fear conditioning, Aging

## Abstract

Chronic exposure to stress during midlife associates with subsequent age-related cognitive decline and may increase the vulnerability to develop psychiatric conditions. Increased hypothalamic-pituitary-adrenal (HPA) axis activity has been implicated in pathogenesis though any causative role for glucocorticoids is unestablished. This study investigated the contribution of local glucocorticoid regeneration by the intracellular enzyme 11β-hydroxysteroid dehydrogenase type 1 (11β-HSD1), in persisting midlife stress-induced behavioral effects in mice. Middle-aged (10 months old) 11β-HSD1-deficient mice and wild-type congenic controls were randomly assigned to 28 days of chronic unpredictable stress or left undisturbed (non-stressed). All mice underwent behavioral testing at the end of the stress/non-stress period and again 6–7 months later. Chronic stress impaired spatial memory in middle-aged wild-type mice. The effects, involving a wide spectrum of behavioral modalities, persisted for 6–7 months after cessation of stress into early senescence. Enduring effects after midlife stress included impaired spatial memory, enhanced contextual fear memory, impaired fear extinction, heightened anxiety, depressive-like behavior, as well as reduced hippocampal glucocorticoid receptor mRNA expression. In contrast, 11β-HSD1 deficient mice resisted both immediate and enduring effects of chronic stress, despite similar stress-induced increases in systemic glucocorticoid activity during midlife stress. In conclusion, chronic stress in midlife exerts persisting effects leading to cognitive and affective dysfunction in old age via mechanisms that depend, at least in part, on brain glucocorticoids generated locally by 11β-HSD1. This finding supports selective 11β-HSD1 inhibition as a novel therapeutic target to ameliorate the long-term consequences of stress-related psychiatric disorders in midlife.

## Highlights

• Spatial memory deficits after midlife stress remain for 6 months.
• Enhanced fear memory and impaired extinction 6 months after midlife stress.
• Increased anxiety and depressive-like behaviors 7 months after midlife stress.
• 11β-HSD1 deficient mice resist enduring effects of midlife stress on behaviors.

## 1 Introduction

Chronic stress, which activates the HPA axis and thus increases blood levels of glucocorticoid (GC) stress hormones, causes contemporary cognitive dysfunction and may contribute to the development of depression and anxiety-related disorders (Lupien et al., 2009; Pryce and Fuchs, 2017). Stress or GC overexposure in early life (prenatal, early postnatal, juvenile) can “programme” lifetime alterations in cognitive and affective function (Meaney et al., 1988; Patchev et al., 2014). Whether an episode of chronic stress in adulthood has persisting effects or interacts with natural aging processes to advance cognitive decline and alter affective behaviors is unclear.

Recent human population-based studies have shown an association between levels of stress and accelerated cognitive decline in adults at 65 years and older (Aggarwal et al., 2014). A longitudinal study found that midlife stress in women was associated decades later with a 60% greater risk of developing dementia (Johansson et al., 2010). Animal studies show that vulnerability to GC or stress-induced cognitive impairment is greater in middle and old age compared with young adults (Bodnoff et al., 1995; Sandi and Touyarot, 2006).

Although mechanisms leading to the development of stress-related cognitive and anxiety-type disorders are not fully understood, GCs have an important role. The hippocampus, with its high expression of corticosteroid receptors [mineralocorticoid receptor (MR) and glucocorticoid receptor (GR)], is particularly vulnerable to chronic stress or high GC levels which reduce dendritic complexity (Magarinos and McEwen, 1995), and alter synaptic plasticity (Kim et al., 2007). GC activation of MR and GR modulates HPA axis and cognitive function (Harris et al., 2013). Moreover, elevated circulating GC levels as a consequence of impaired HPA axis negative feedback correlate with impaired hippocampal dependent memory performance in aged humans and animals (Issa et al., 1990; Lupien et al., 1998).

It is now clear that active GCs (corticosterone in rodents) within specific brain regions are derived not only from the circulation (only ∼5% of circulating active GCs are unbound and thus available to penetrate the blood brain barrier), but also by local regeneration from inert and largely unbound circulating 11-keto forms (11-dehydrocorticosterone). This local regeneration is catalysed by the intracellular enzyme, 11β-hydroxysteroid dehydrogenase type 1 (11β-HSD1) which is abundantly expressed in brain regions involved in cognition, anxiety and HPA axis regulation including the hippocampus, cortex and amygdala (Wyrwoll et al., 2011). Importantly, increased brain GC levels derived locally from 11β-HSD1 activity have emerged as key determinants of age-related cognitive decline (Yau and Seckl, 2012; Yau et al., 2015a). In wild type mice, 11β-HSD1 mRNA expression in the hippocampus and cortex increases with aging and correlates with impaired spatial memory (Holmes et al., 2010). Young adult 11β-HSD1 knockout mice show normal performances in spatial memory and anxiety behaviors (Yau et al., 2007; Yau et al., 2001). However, a protective cognitive phenotype becomes apparent with aging in 11β-HSD1 knockout mice (Yau and Seckl, 2012). Thus, 11β-HSD1 knockout mice resists age-related cognitive deficits found in aged matched wild type mice (Yau et al., 2007; Yau et al., 2001). Short-term administration of selective 11β-HSD1 inhibitors, which lower intra-hippocampal GC levels without affecting circulating GC levels, improves spatial memory in already aged mice (Sooy et al., 2010; Wheelan et al., 2015; Yau et al., 2015a). Any role of 11β-HSD1 deficiency to modulate the impact of chronic stress on cognitive and affective functions is unexplored.

We investigated whether a period of chronic stress in midlife has long lasting detrimental effects on cognition, anxiety and mood-related behaviors into early old age in mice. Psychological ‘rat predator’ stressors were included in a chronic unpredictable stress paradigm that models the uncontrollable nature of adverse life events (Herman et al., 1995). We also examined if these effects are prevented or attenuated by 11β-HSD1 deficiency.

## 2 Material and methods

### 2.1 Animals

Mice homozygous for targeted disruption of the *Hsd11b1* gene that encodes 11β-HSD1 (*Hsd11b1^−/−^*, KO), congenic on the C57BL/6J genetic background (Carter et al., 2009), and age-matched C57BL/6J wild type (*Hsd11b1^+/+^*, WT) controls were bred in house. The C57BL/6J genetic background chosen is the most widely used inbred mouse strain for behavioral testing. All mice were housed under standard conditions (7:00 am to 7:00 pm light/dark cycle, 21° C) with food and water available *ad libitum* until experimentation at ∼10 months of age. Animal procedures were carried out under the auspices of the UK Animals (Scientific Procedures) Act 1986 and the European Communities Council Directive of 22 September 2010 (Directive 2010/63/EU).

### 2.2 Midlife chronic variable stress

Male WT and KO mice at ∼10 months of age (midlife) were randomly assigned to non-stress or stress groups (n = 10/group/genotype). The chronic stress paradigm was identical for WT and KO mice, with stressors applied to the two groups concurrently, and consisted of exposure to one or two alternating stressors daily 6 days per week for 4 weeks. Stressors applied included (1) restraint in small rodent plastic restraint tubes for 2 min, 5 min, 10 min or 15 min; (2) forced warm swim (25° C or 30° C) for 1 min, 2 min or 3 min; (3) forced cold swim (15° C) for 1 min or 2 min; (4) non-contact predator exposure (rat) for 4 h or overnight; (5) predator odour exposure (rat soiled bedding material) overnight; (6) overnight lighting; (7) removal of nesting material overnight; (8) novel object placement within home cages overnight and (9) isolated housing for 4 h. The timing and duration of the morning or afternoon stressors and the day of the week with no stress exposure varied in an unpredictable manner. Non-stressed mice were transported to/from procedure rooms in their home cages but were not handled.

### 2.3 Behavioral testing

All mice underwent behavioral testing in a longitudinal manner starting the day after the last stressor in midlife with spatial recognition memory (Y-maze) followed by contextual fear memory. After a rest period of 6 months, the following tests were carried out in the order listed: spatial memory (Y-maze), contextual fear memory, anxiety-related behaviors (open field and elevated plus maze), spatial working memory (Y-maze) and depressive-like behavior (tail suspension test). For a detailed timeline of stress exposure and behavioral testing from midlife to early aging (18 months) see supplementary information (Fig. S1). To minimize the impact of each behavioral task affecting the outcome of later testing with carryover effects, the order of testing was from least stressful (Y-maze) to more stressful (tail suspension) with a rest period of 2–7 days between different tasks. The affective behavior tasks were carried out only in early old age (and not midlife) as these are highly influenced by earlier testing.

Mice were transported to the behavior testing room in their home cages and left to acclimatize for at least 30 min prior to behavioral assessment. The movement of each mouse in the test apparatus (Y-maze, elevated-plus maze and open field) was tracked using a ceiling mounted camera and analysed using ANYMAZE software (Stoelting, Dublin, Ireland).

#### 2.3.1 Y-maze

Spatial recognition and working memories were tested in a Y-maze surrounded with external cues to aid navigation as described previously (Yau et al., 2007). Y-maze navigation is based on the animal’s innate curiosity to explore novel areas and is not considered stressful. For spatial recognition memory, mice were placed into one of the arms of the maze (start arm) and allowed to explore the maze for 5 min with the entrance of one arm blocked off (training trial). After a 2 h inter-trial interval (ITI), mice were returned into the start arm of the maze for the test phase with all three arms available for exploration (5 min) including the novel (previously unvisited) arm. Spatial memory retention was measured as time spent in the novel arm calculated as a percentage of the total time spent in all three arms. The external cues surrounding the Y-maze were changed for re-testing 6 months later in aged mice. For spatial working memory (spontaneous alternation) mice were placed in the centre of the Y-maze and allowed to explore all three arms for 5 min. The number of arm entries and the sequence of arms entered were recorded. The percentage alternation was calculated as number of alternations (entries into three different arms consecutively) divided by the total possible alternations (i.e. number of arms entered minus 2) and multiplied by 100.

#### 2.3.2 Open-Field test

The apparatus and experimental conditions for open-field testing were as previously described (Yau et al., 2007). Each mouse was placed in the centre of the open field arena and allowed to explore for 5 min. Times spent in the centre and at the sides of the apparatus were scored.

#### 2.3.3 Elevated plus-maze (EPM)

The apparatus and experimental conditions used for EPM were as described (Yau et al., 2007). Each mouse was placed in the central platform of the maze facing one of the open arms and allowed to explore freely for 5 min. Times spent in the open and enclosed arms were scored.

#### 2.3.4 Tail suspension test (TST)

Each mouse was suspended 30 cm off the floor by the base of the tail taped to the edge of a table with its body dangling in the air. The TST sessions were recorded with a digital handheld camera and lasted 5 min. Mice initially struggle to face upward but then stop struggling and hang immobile. The time spent immobile, indicative of a depressive-like behavior (Steru et al., 1985), was scored for each mouse by two observers blind to the experimental conditions.

#### 2.3.5 Fear conditioning

A classical fear-conditioning (FC) paradigm, in which a novel environment (non-aversive conditioned stimulus) within a FC enclosure (Coulbourn Instruments, Whitehall PA) was paired with an aversive unconditioned stimulus (electric footshock), was conducted essentially as previously described (Wheelan et al., 2015). Briefly, mice were observed during an acclimatisation session (4 min) in the FC enclosure (neutral context − silver aluminium tiles on walls) to assess baseline levels of freezing. On the following day, mice were fear-conditioned within Context A (patterned wall tiles and visual spatial cues) with a single electric foot shock (2 s, 0.6 mA). Contextual fear memory was assessed 24 h later when mice were re-exposed to conditioning Context A but without foot shock. The freezing response, defined as the absence of all movements except respiration, was measured for 4 min and analysed using FreezeFrame software (Actimetrics, Illinois, USA).

Six months later, mice underwent another fear conditioning training with exposure to a novel Context B for 3 min followed by two foot shocks (2 s, 0.6 mA) separated by 30 s. One day after conditioning, mice were placed back into Context B for 4 min without foot shocks in order to extinguish fear responding to the context. Further extinction trials (Context B exposure without foot shocks) were carried out over the next 4 consecutive days and again 7 days later to confirm extinguished conditioned fear responses. Four hours later, mice were exposed to Context B paired with a single sub-threshold reminder shock (1 s, 0.3 mA), and on the following day, re-exposed to Context B (no foot shock) to measure reinstatement of fear memory after extinction.

### 2.4 Physiological measures

Basal tail venesection blood was sampled (i) between 8.00 and 10.00 A.M. before chronic stress, (ii) at the end of chronic stress and (iii) 6 months later. For 24 h measurements, fecal pellets were collected daily for 2 days from all groups 6.5 months after stress cessation into pre-weighed tubes and stored at −20 °C until analysed. Fecal steroids were extracted by a method using dichloromethane as previously described (Morton et al., 2005). Plasma and fecal corticosterone (CORT) levels were measured using in-house radioimmunoassay and ELISA assays, respectively, as previously described (Al-Dujaili et al., 2009; Harris et al., 2001).

All mice were weighed prior to chronic stress (10 months old), then weekly during chronic stress and finally at the end of the experiment (18 months old). One week following the tail suspension test, all mice were killed in the morning by cervical dislocation and brains removed, frozen on powdered dry ice and stored at −80 °C. Thymus and the right adrenal glands were removed and weighed.

### 2.5 In situ hybridization histochemistry

GR, MR and brain-derived neurotrophic factor (*Bdnf*) mRNAs were measured at the level of dorsal hippocampus in cryostat brain sections by *in situ* hybridization histochemistry as previously described (Caughey et al., 2017). Sections were hybridised with ^35^S-UTP labelled RNA antisense probes complementary to GR, MR and *Bdnf*. Following ribonuclease A treatment and a series of standard saline citrate washes, slides were exposed to autoradiographic film and later dipped in photographic emulsion (NTB-2; Kodak, Rochester, NY, USA) for silver grain analysis as previously described (Caughey et al., 2017).

### 2.6 Statistical analysis

All data (except body weights and fear extinction) were analyzed with GraphPad Prism version 6.0 h using two-way analysis of variance (ANOVA) with stress (control vs. stress) and genotype [wild-type (WT) vs. *Hsd11b1^−/−^* (KO)] as independent variables. Body weights and fear extinction data were normally distributed, had equal variance and were analysed using JMP 13.1 for Macintosh (SAS, NC, USA) using repeated measures 2-way ANOVA with stress and genotype as main effects and day as a repeated measure. The Greenhouse-Geisser correction was used to correct for any violations of sphericity (resulting in adjustment of degrees of freedom to non-whole integers). Statistical differences were determined with Bonferroni’s multiple comparisons *post hoc* test (adjusted *p*-values are shown where appropriate) or with Tukey’s HSD *post hoc* test for repeated measures ANOVA. All significant (p < 0.05) main effects and interactions are reported. Data are presented as the mean ± SEM.

## 3 Results

### 3.1 11β-HSD1 deficiency protects against chronic stress-induced spatial memory deficits but impaired contextual fear memory in middle-aged mice

**Fig. 1:**
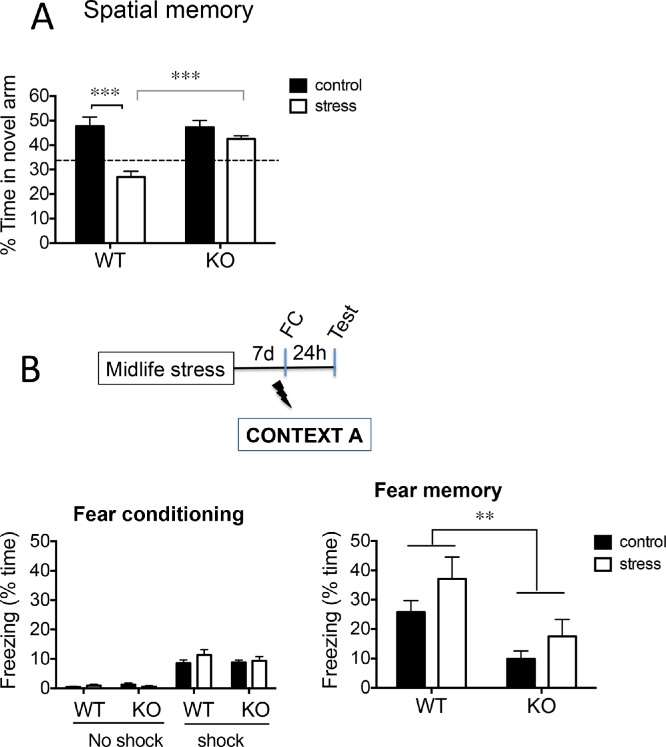
11β-HSD1 deficiency protect against chronic stress-induced spatial memory deficits but impaired contextual fear memory in middle-aged mice. (A) Chronic stress in WT (but not KO) mice leads to impaired spatial memory (less time in novel arm after 2 h ITI in Y-maze compared to non-stressed WT controls). Dotted line represents chance performance levels. (B) Timeline of fear conditioning (FC) to context A (paired with single 0.6 mA footshock) carried out 7 d after midlife stress. Chronic stress had no affect on contextual fear memory (freezing responses to context A exposure without foot shock 24 h after FC) in WT or KO mice. However, KO mice (both stressed and non-stressed controls) show reduced freezing responses compared to corresponding WT mice. Bonferroni post hoc tests, ** p < 0.01, ***p < 0.001, n = 8-10/group. Data shown are mean ± SEM.

Spatial recognition memory was determined in the Y-maze one day after the end of chronic stress in middle-aged mice. Non-stressed WT and KO mice showed similar intact spatial memory, spending significantly more time in the novel arm than already explored arms as previously described (Yau et al., 2007). Analysis of the percentage time spent in the novel arm revealed that genotype effects were evident only under the stress condition [2-way ANOVA; stress: F_1,32_ = 25.1, p < 0.0001; genotype: F_1,32_ = 8.9, p < 0.01; stress x genotype interaction: F_1,32_ = 9.9, p < 0.01]. Chronic stress impaired spatial memory in WT mice as indicated by 43% decrease in time spent in novel arm (p < 0.001) compared to non-stressed WT controls and at chance levels (Fig. 1A). In contrast, chronic stress had no significant effect on spatial memory in KO mice (time spent in novel arm was greater (p < 0.001) in stressed KO than stressed WT mice; Fig. 1A). 11β-HSD1 deficiency *per se* had no effect on spatial memory in non-stressed control mice (WT vs KO, Fig. 1A).

Contextual fear memory, measured 24 h after fear conditioning (Context A with foot shock), was not significantly affected by stress in WT or KO mice [F_1,33_ = 3.4, p = 0.07] as shown by freezing response to Context A alone (Fig. 1B). However, there was an effect of genotype [F_1,33_ = 12, p < 0.01] but no interaction with stress (p = 0.72). KO mice showed significantly impaired fear memory compared to corresponding WT mice regardless of chronic stress exposure (Fig. 1B).

### 3.2 Chronic stress in midlife leads to spatial memory deficits, enhanced fear memory, impaired extinction and post-extinction fear reinstatement in aged wild-type (WT) but not *Hsd11b1*^*−/−*^(KO) mice

#### 3.2.1 Spatial recognition and working memories

**Fig. 2:**
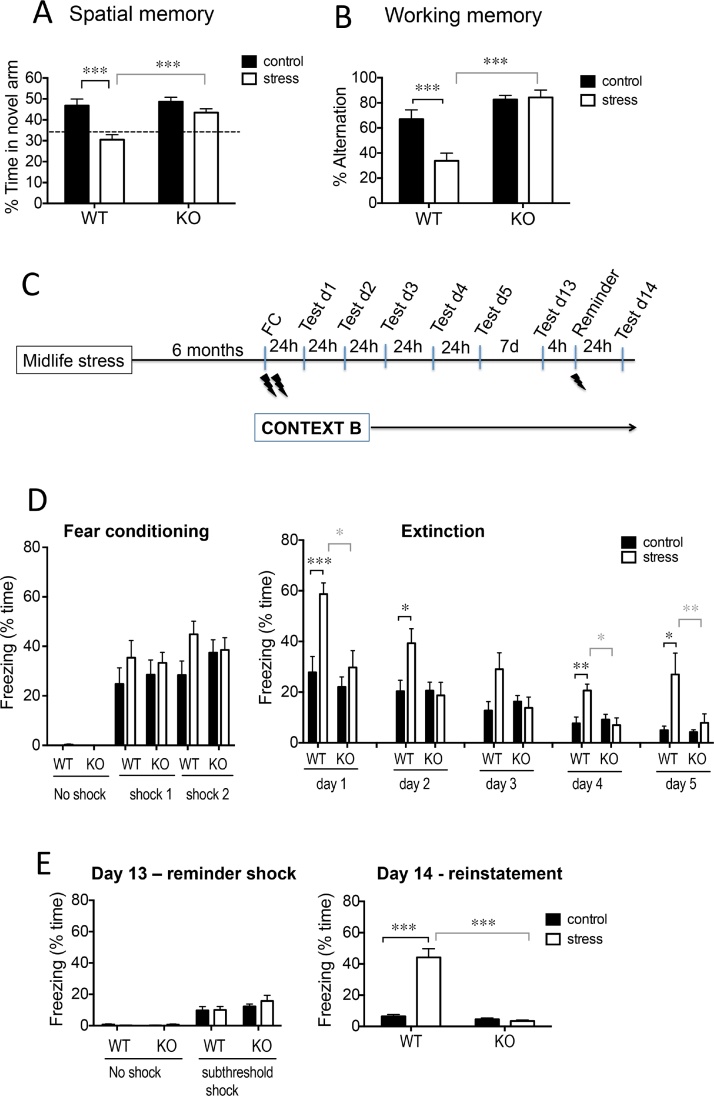
Chronic stress in midlife leads to spatial memory deficits, enhanced fear memory, impaired extinction and post-extinction fear reinstatement in aged wild-type (WT) but not *Hsd11b1^−/−^* (KO) mice. (A) Spatial memory remains impaired (less time in novel arm after 2 h ITI in Y-maze) in aged (17 months) WT but not KO mice 6 months after midlife stress. Dotted line represents chance performance levels. (B) Midlife chronic stress impaired working memory (% alternation in arms of Y-maze) in aged WT but not KO mice 7 months after stress cessation. (C) Timeline of fear conditioning (FC) (Context B paired with 2 × 0.6 mA foot shocks) and extinction tests (exposure to Context B with no foot shocks). A single subthreshold reminder 0.3 mA foot shock 7 days post extinction measured reinstatement of extinguished fear. (D) Midlife chronic stress enhanced contextual fear responses and impaired fear extinction in aged WT but not KO mice. 11β-HSD1 deficiency had no effect on fear extinction in non-stressed control mice. (E) Aged WT mice with history of midlife stress (but not KO mice) show fear memory reinstatement 24 h after exposure to a reminder shock. Bonferroni or Tukey’s HSD *post hoc* tests, *p < 0.05, ** p < 0.01, *** p < 0.001, n = 9–10/group. Data shown are mean ± SEM.

Spatial recognition memory remained impaired in 17 months old WT mice with prior midlife stress experience as indicated by less time in novel arm, P < 0.001 compared to non-stressed WT and stressed KO mice [2-way ANOVA; stress: F_1,33_ = 20.0, p < 0.0001; genotype: F_1,33_ = 9.5, p < 0.01; and stress x genotype interaction: F_1,33_ = 5.4, p < 0.05; Fig. 2A]. In contrast, spatial memory was not impaired in aged KO mice previously stressed in midlife [% time in novel arm was greater (p < 0.001) than corresponding stressed WT mice, Fig. 2A]. 11β-HSD1 deficiency had no effect on spatial memory in non-stressed control mice (WT vs KO, Fig. 2A).

Working memory (% alternation in arms of Y-maze) was measured 7 months after cessation of midlife stress. Prior midlife stressed aged WT mice showed impaired working memory as indicated by less% alternation, p < 0.001 compared to non-stressed WT controls and stressed KO mice [2-way ANOVA; stress: F_1,34_ = 7.3, p < 0.05; genotype: F_1,34_ = 32.0, p < 0.0001; and stress x genotype interaction: F_1,34_ = 8.8, p < 0.01; Fig. 2B]. In contrast, working memory was not affected (p > 0.99) by midlife stress in aged KO mice (Fig. 2B). 11β-HSD1 deficiency had no effect on working memory among non-stressed control mice (WT vs KO: p = 0.13, Fig. 2B).

#### 3.2.2 Contextual fear memory extinction

Contextual fear conditioning 6 months after midlife stress (Fig. 2C), resulted in post-shock freezing responses which were ∼ 2 fold greater than responses in middle-age, regardless of stress experience in both WT and KO mice (Figs. Figure 1B and Figure 2D). During fear extinction, prior midlife stressed WT mice displayed higher levels of freezing compared to non-stressed WT controls, but stress experience had no impact on freezing in KO mice [2-way repeated-measures ANOVA; stress: F_1,34_ = 9.8, P < 0.01; genotype: F_1,34_ = 6, p < 0.05; stress x genotype: F_1,34_ = 4.9, p < 0.05; day x stress: F_2.7,92.7_ = 5.1, p < 0.01; Fig. 2D]. Freezing levels decreased over the days in all groups (day: F_2.7,92.7_ = 56.5, p < 0.0001, Fig. 2D). There were no other significant interactions. Extinction was impaired in aged WT mice with history of midlife stress as shown by ∼31% decrease in freezing from day 2–5 compared with ∼77% decrease in non-stressed WT mice (Fig. 2D). Fear memory extinction in aged KO mice (∼75.5% reduction in freezing responses from day 2–5; Fig. 2D) was similar to non-stressed WT mice. Interestingly, the reduced fear memory associated with 11β-HSD1 deficiency observed in middle-aged mice (Fig. 1B) was not observed in aged KO mice 24 h after conditioning with two foot shocks (Fig. 2D, day 1).

#### 3.2.3 Post extinction contextual fear memory reinstatement

Seven days after the extinction trial on day 5, mice were exposed to the same Context B and given a subthreshold foot shock (equivalent to re-exposure to mild trauma) to examine if this can reinstate an extinguished fear memory (Fig. 2C). Prior to the shock, aged WT and KO mice showed no freezing responses, confirming extinguished fear memory and no spontaneous recovery. WT mice with prior stress experience showed significantly enhanced fear memory recovery 24 h after the reminder shock [∼7 fold increase in freezing (p < 0.001) compared to non-stressed WT controls and stressed KO mice; 2-way ANOVA; stress: F_1,36_ = 40.1, p < 0.0001; genotype: F_1,36_ = 53.9, p < 0.0001; stress x genotype: F_1,36_ = 44.9, p < 0.0001, Fig. 2E]. In contrast, KO mice did not show significant fear memory recovery (24 h after reminder shock) regardless of prior stress experience (Fig. 2E).

### 3.3 11β-HSD1 deficiency protects against effects of midlife chronic stress on affective behaviors in aged mice

#### 3.3.1 Anxiety-like behaviors

**Fig. 3:**
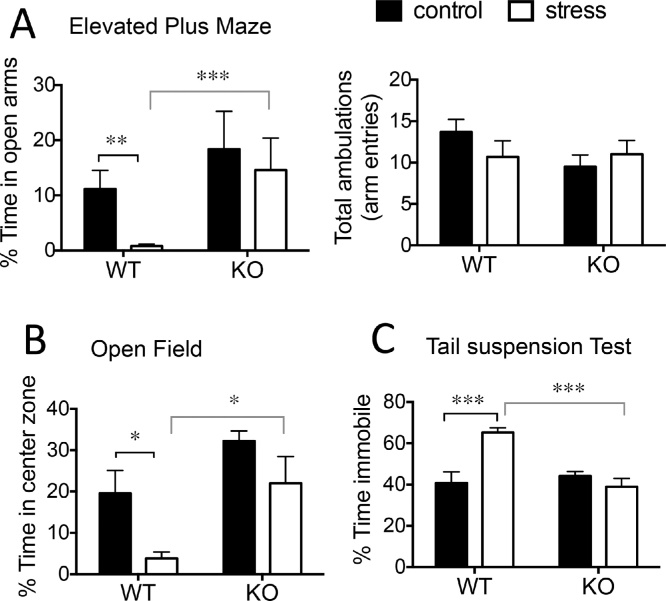
11β-HSD1 deficiency protect against effects of midlife chronic stress on affective behaviors in aged mice. Midlife stress increased anxiety [reduced time in open arms of EPM (A); decreased time in centre zone of open field (B)] and depressive-like behavior [increased immobility in tail suspension test (C)] in aged WT but not KO mice 6 months after stress cessation. Total ambulation (arm entries) in EPM, measured as an indicator of locomotor activity, did not differ between groups (A). Bonferroni post hoc tests, *p < 0.05, *** p < 0.001, n = 8–10/group. Data shown are mean ± SEM.

Aged WT mice exposed to midlife stress were more anxious compared to non-stressed WT mice (and midlife stressed KO mice) as indicated by less time spent on the open arms of the EPM [2-way ANOVA; stress: F_1,30_ = 7.1, p < 0.05; genotype: F_1,30_ = 11.7, p < 0.01; stress x genotype interaction: F_1,30_ = 9.0, p < 0.01] and less time spent in the centre of the open field [2-way ANOVA; stress; F_1,32_ = 8.3, p < 0.01; genotype: F_1,32_ = 11.7, p < 0.01] (Fig. 3A and B). This was not caused by differences in overall activity since total ambulations (arm entries) were similar between groups [2-way ANOVA; stress: F_1,37_ = 0.2, p = 0.65; genotype: F_1,37_ = 1.4, p = 0.24]; Fig. 3A. Aged KO mice, showed no effect of prior midlife stress experience on anxiety-related behaviors in both EPM and open field tests, (Fig. 3A and B). Anxiety-related behaviors exhibited in non-stressed groups were not significantly affected by a deficiency of 11β-HSD1 [time spent in open arms of EPM: WT vs KO, p = 0.9, Fig. 2A; centre of open field: WT vs KO, p = 0.14, Fig. 2B].

#### 3.3.2 Depressive-like behavior

Depressive-like behavior in the aged mice, as indicated by immobility in the tail suspension test (TST), was significantly affected by midlife stress [2-way ANOVA; stress: F_1,31_ = 6.4, p < 0.05; genotype: F_1,31_ = 9.0, p < 0.01; stress x genotype interaction: F_1,31_ = 15.2, p < 0.001]. Aged WT mice with history of prior midlife stress showed increased immobility (p < 0.001) compared to non-stressed WT mice and stressed KO mice (Fig. 3C). In contrast, aged KO mice showed no effect of prior midlife stress exposure on immobility (Fig. 3C). Immobility among non-stressed groups was not significantly affected by a deficiency of 11β-HSD1 [time spent immobile: WT vs KO, p = 0.78, Fig. 3C].

### 3.4 Effect of 11β-HSD1 deficiency and midlife chronic stress exposure on physiological measures in middle-aged and aged mice

#### 3.4.1 Body weights

**Fig. 4:**
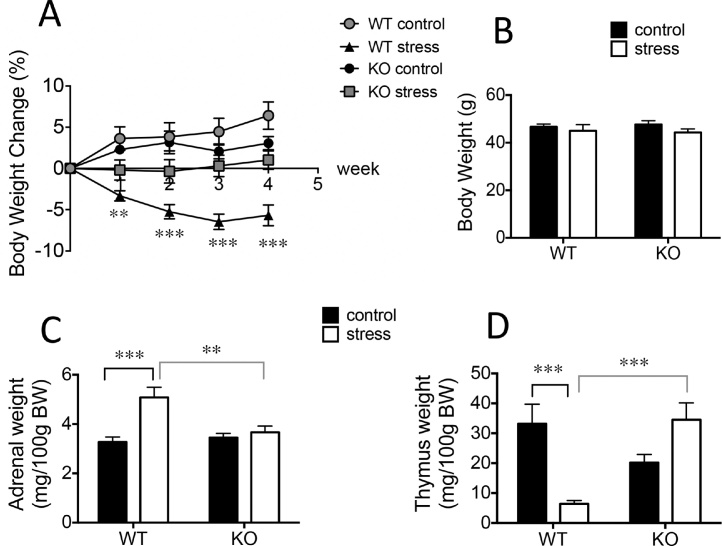
Effect of 11β-HSD1 deficiency and midlife chronic stress exposure on physiological measures in middle-aged and aged mice. (A) Chronic stress decreased body weight change (expressed as percentage of initial weight before stress exposure) in WT mice (compared to non-stressed WT controls) for the 4-week duration. KO mice do not gain or lose body weight during the period of stress. (B) Body weight changes recover seven months after cessation of stress. (C) Chronic stress in midlife increased adrenal weights in aged WT but not KO mice seven months after stress cessation. (D) Midlife chronic stress decreased thymus weights in aged WT but not KO mice seven months after cessation of stress. 11β-HSD1 deficiency had no significant effect on thymus weights in non-stressed aged control mice (WT vs KO). Bonferroni post hoc tests, **p < 0.01, ***p < 0.001, n = 9–10/group. Data shown are mean ± SEM.

Stress decreased body weight gain [2-way repeated-measures ANOVA; main effect of stress: F_1,37_ = 35.7, p < 0.0001], but only in WT mice [stress x genotype x week: F_3.2,118.9_ = 5.0, p < 0.01]. Stressed WT mice lost weight over the course of the 4-week period compared to non-stressed WT and KO mice which gained weight, but KO stressed mice did not gain or lose body weight (Fig. 4A; Post hoc Tukey HSD, p < 0.05). Body weights of aged mice were not affected by genotype or midlife stress exposure (Fig. 4B).

#### 3.4.2 Adrenal and thymus weights

Adrenal mass was significantly affected by stress in the aged mice [2-way ANOVA; stress: F_1,33_ = 12.4, p < 0.01; genotype: F_1,33_ = 4.6, p < 0.05; stress x genotype interaction: F_1,33_ = 7.7, p < 0.01]. Aged WT mice with prior midlife stress exposure showed heavier adrenals compared to non-stressed WT controls (p < 0.001) and stressed KO mice (p < 0.01; Fig. 4C). For thymus mass, there was no overall main effect of stress or genotype but there was a significant stress x genotype interaction [2-way ANOVA; stress: F_1,31_ = 1.8, p = 0.19; genotype: F_1,31_ = 2.6, p = 0.11; stress x genotype interaction: F_1,31_ = 19.6, p < 0.001]. Thymus weights were decreased by prior midlife stress exposure in aged WT compared to non-stressed WT controls (p < 0.001) and stressed KO mice (p < 0.001), Fig. 4D. These alterations in adrenal and thymus weights observed in midlife stressed aged WT mice suggest greater glucocorticoid secretion and effects upon tissues susceptible to their trophic actions (Fig. 4C and D). Adrenal and thymic weights were not affected by stress exposure in KO mice (p > 0.9 and p = 0.08, respectively, Fig. 4C and D). 11β-HSD1 deficiency had no significant effect on adrenal (WT vs KO, p > 0.9) and thymus weights (WT vs KO, p = 0.12) in non-stressed mice (Fig. 4C and D)

### 3.5 Effect of 11β-HSD1 deficiency and midlife chronic stress exposure on CORT levels in middle-aged and aged mice

**Fig. 5:**
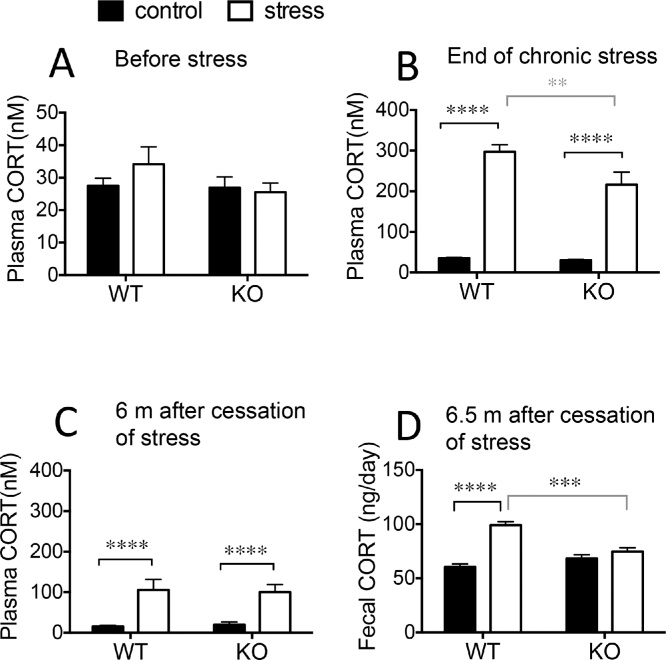
Effect of 11β-HSD1 deficiency and midlife chronic stress exposure on CORT levels in middle-aged and aged mice. (A) Basal plasma CORT levels in WT and KO middle-aged mice before chronic stress. (B) Elevated plasma CORT levels the morning after the last stressor in WT and KO middle-aged mice. (C) Plasma CORT levels remain elevated 6 months following cessation of chronic stress in both WT and KO aged mice. (D) Fecal CORT levels remain elevated in aged WT but not KO mice 6.5 months following cessation of chronic stress. Bonferroni post hoc tests, **p < 0.01, ***p < 0.001, **** p < 0.0001, n = 9–10/group. Data shown are mean ± SEM.

Before starting stress treatment, basal morning plasma CORT levels were similar in WT and KO mice (Fig. 5A). At the end of chronic stress, plasma CORT levels were significantly elevated in both genotypes (Fig. 5B) but with higher stressed levels in WT than KO mice [2-way ANOVA; stress: F_1,35_ = 176.6, p < 0.0001; genotype: F_1,35_ = 6.6, p < 0.05; stress x genotype interaction: F_1,35_ = 5.0, p < 0.05]. Six months later in senescence, plasma CORT levels were modestly elevated in both stressed WT and KO mice (Fig. 5C) compared with age-matched, non-stressed mice [2-way ANOVA: main effect of stress: F_1,36_ = 27.9, p < 0.0001]. Integrated CORT levels measured in fecal pellets collected over 2 days were elevated in aged WT mice with a history of midlife stress [2-way ANOVA; stress: F_1,14_ = 43.8, p < 0.0001; genotype: F_1,14_ = 5.9, p < 0.05; stress x genotype interaction: F_1,14_ = 22.2, p < 0.001] (Fig. 5D). Fecal CORT levels in aged KO mice were not affected by prior midlife stress and were similar to non-stressed aged WT mice (Fig. 5D).

### 3.6 Midlife chronic stress leads to decreased hippocampal GR mRNA expression in aged WT but not 11β-HSD1 deficient mice

**Fig. 6:**
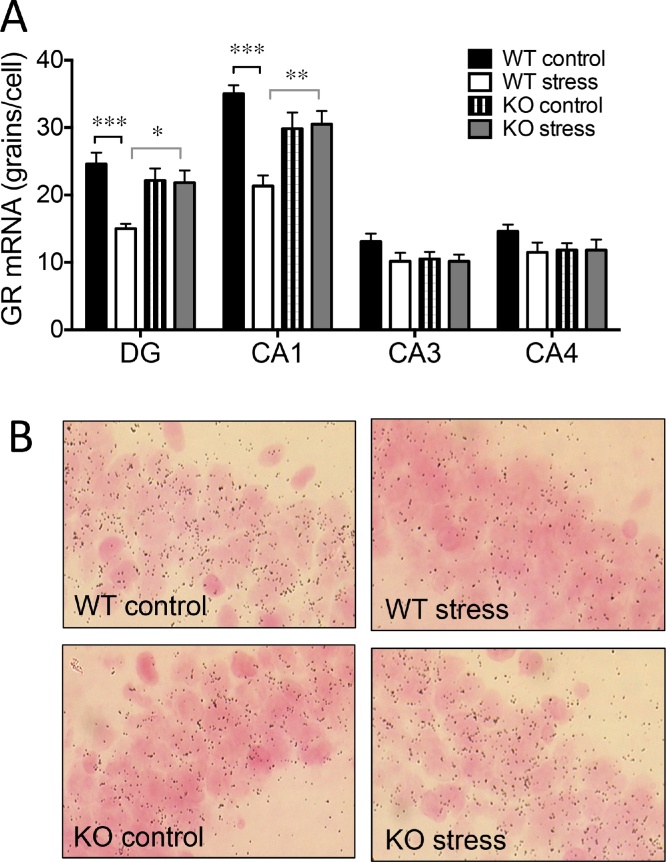
Midlife chronic stress leads to decreased hippocampal GR mRNA expression in aged WT but not 11β-HSD1 deficient mice. (A) Chronic stress in midlife reduced GR mRNA expression in dentate gyrus (DG) and CA1 subregion of the hippocampus in aged WT but not KO mice. (B) Representative photomicrographs of *in situ* hybridization under bright-field illumination showing GR mRNA expression in CA1 subregion of the hippocampus from aged WT and KO mice 7 months after cessation of midlife stress and corresponding non-stressed controls. Bonferroni post hoc tests, *p < 0.05, **p < 0.01, ***p < 0.001, n = 10/group. Silver grains appear black. Data shown are mean ± SEM.

Hippocampal GR mRNA expression was not affected by 11β-HSD1 deficiency in aged mice (Fig. 6A) as previously reported (Yau et al., 2011). Midlife stress caused a significant decrease in GR mRNA levels 7 months later in the dentate gyrus and CA1 subregion of the hippocampus in aged WT but not 11β-HSD1 KO mice [2-way ANOVA: stress in DG: F_1,19_ = 10.0, p < 0.01; stress in CA1: F_1,19_ = 11.6, p < 0.01; stress x genotype interaction in DG: F_1,19_ = 8.7, p < 0.01; stress x genotype interaction in CA1: F_1,19_ = 14.2, p < 0.01]; (Fig. 6A and B). MR and *Bdnf* mRNA expression were unaffected by 11β-HSD1 deficiency or midlife stress exposure in the hippocampus of aged mice (Supplementary information, Fig. S2).

## 4 Discussion

Chronic unpredictable stress exposure during midlife in WT mice led to (i) impaired spatial memory, an effect that persisted 6 months beyond stress recovery to advance age-related cognitive decline, and (ii) the emergence of abnormal potentiated fear memory together with enhanced anxiety- and depressive-like behaviors in early old age. 11β-HSD1-deficient mice resisted these effects suggesting an important contribution of local 11β-HSD1-generated GCs in the enduring stress-induced alterations in cognitive and affective behaviors.

### 4.1 Midlife chronic stress: immediate and long-lasting effects on spatial memory

Chronic stress exposure in young adult animals has predominantly transient behavioral and structural brain effects (Magarinos and McEwen, 1995; McEwen and Gianaros, 2011) including impaired hippocampus-dependent cognition (Cordner and Tamashiro, 2016; Kim et al., 2007). However, our study show that midlife chronic stress-induced spatial memory deficits persisted for 6 months after stress cessation into early old age in WT mice suggesting the normally reversible effects of chronic stress is compromised with aging. Similarly, middle-aged rats fail to show reversal of stress-induced morphological plasticity in prefrontal neurons that was observed in young adult rats (Bloss et al., 2010). One previous study also showed impaired spatial memory 6 months after midlife chronic stress but only in a subgroup of aged rats with high reactivity to novelty (Sandi and Touyarot, 2006).

### 4.2 Midlife chronic stress effects on contextual fear memory

Contextual fear memory, which depends on functional integrity of the hippocampus and amygdala, was not significantly enhanced in middle-aged WT mice days after cessation of chronic stress, in contrast with previous findings in young adult rats (Hoffman et al., 2015; McGuire et al., 2010). The non-significant (p = 0.07) trend for chronic stress enhanced fear memory in the WT mice suggest that this discrepancy may be due, in part, to differences in fear conditioning protocols with the current study using a single foot shock compared to three foot-shocks in the rat studies. However, enhanced contextual fear memory was observed 6 months later after midlife stress in line with reports in young adult rats 16 weeks after predator stress (Zoladz et al., 2015). This more pronounced fear memory with the passage of time following midlife stress, together with the initial fear conditioning experience, is consistent with conditioned fear incubation (Pickens et al., 2009) and mimics one critical aspect of delayed-onset PTSD in humans (Andrews et al., 2007). While studies have shown impaired fear memory extinction when tested days after stress (Hoffman et al., 2014; Izquierdo et al., 2006), we show for the first time impaired fear extinction at least 6 months after a period of midlife stress in aged WT mice.

Fear extinction does not “erase” the previously learned context-shock association but is thought to be a form of inhibitory learning of the acquired fear (Myers and Davis, 2007). Indeed, a sub-threshold reminder shock after fear extinction caused a significant recovery of conditioned fear to context 24 h later in midlife stressed aged WT mice. The lack of fear reinstatement in non-stressed aged WT mice suggest that chronic stress exposure may increase the likelihood of recovery of an extinguished fear.

### 4.3 Midlife chronic stress effects on anxiety and depressive-like behaviors in early old age

In addition to impaired spatial memory, enhanced fear memory and impaired extinction, aged WT mice also showed increased anxiety and depressive-like behaviors 6 months after midlife stress, consistent with the persistent behavioral abnormalities associated with stress-related psychopathologies (Finsterwald and Alberini, 2014). Whereas increased anxiety and depressive-like behaviors have been reported days after chronic stress in rats and mice (Bhagya et al., 2017; Santana et al., 2015), few studies have examined affective behaviors months after stress. Increased anxiety-related behaviors have been reported in rats 3 months after predator stress (Zoladz et al., 2015) but until now the persistence of depressive-like behaviors months after stress cessation has not been described.

### 4.4 Midlife chronic stress effects on physiological measures

In the limited number of studies examining the long-term effects of stress, plasma CORT levels were unaltered (Sandi and Touyarot, 2006; Zoladz et al., 2015) or reduced (Zoladz et al., 2012) following stress recovery. Abnormally low baseline cortisol levels and increased sensitivity to HPA axis negative feedback tend to associate with PTSD in young individuals following trauma (Yehuda and Seckl, 2011) but not all studies show this. The elevated CORT levels (plasma and fecal) in aged WT mice months after midlife stress may reflect the interaction between age and trauma. Indeed, increased cortisol concentrations have been reported in hair of severely traumatized individuals with PTSD (Steudte et al., 2011). Sustained effects on adrenal and thymus mass in WT mice 7 months after midlife stress are also indicators of HPA activation. These effects are consistent with stress-induced adrenal hypertrophy (McGuire et al., 2010; Zoladz et al., 2012) and GC-dependent thymic atrophy (Ulrich-Lai et al., 2006). Whether midlife stressed WT mice show altered HPA axis negative feedback regulation remain to be determined.

### 4.5 11β-HSD1 deficiency protect against midlife chronic stress effects on cognition and affective behaviors

11β-HSD1 deficient mice exposed to midlife chronic stress had similar elevated morning plasma CORT levels to stressed WT mice but failed to show the spatial memory deficits, abnormal fear memory and enhanced affective behaviors. Normally, elevated plasma CORT levels would be anticipated to impair cognition but aged 11β-HSD1 deficient mice show significantly lower increases in intrahippocampal CORT levels during spatial memory acquisition and retrieval in the Y-maze (Yau et al., 2015a). While plasma CORT levels remained modestly elevated in 11β-HSD1 deficient mice 7 months after midlife stress, fecal CORT levels were not altered. This discrepancy in data is not unusual because fecal CORT levels represents a non-invasive integrated measure of free hormone over considerable time (∼24 h), whereas the morning tail nick blood sample is a single “snapshot” of total CORT (both free and bound) that involves handling the mice. Thus, fecal CORT levels may be a better indicator of basal 24 h CORT concentrations. Indeed, aged 11β-HSD1 deficient mice resisted the midlife stress-induced changes in adrenal and thymus weights found in WT mice.

Middle-aged 11β-HSD1 deficient mice showed less robust fear memory compared to WT mice after fear conditioning with a single foot shock, regardless of stress experience, similar to findings in mice following short-term 11β-HSD1 inhibition (Sarabdjitsingh et al., 2014; Wheelan et al., 2015). It is anticipated that lower brain CORT levels in KO mice due to lack of 11β-HSD1 regenerated CORT, even after the single foot shock, may underlie the attenuated fear response compared to WT mice (Roozendaal et al., 1996). Interestingly, when non-stressed control mice were fear-conditioned again 6 months later in a new “context B”, this time with two foot-shocks, the effect of 11β-HSD1 deficiency on fear memory was lost. Although the reason for this is unclear, mice retain contextual memories for at least 60 days (Balogh et al., 2002). Perhaps re-conditioning with two shocks and previous single shock experience may, in combination, increase brain CORT in 11β-HSD1 deficient mice to levels comparable with those required for GR activation to mediate increased fear memory in WT mice (Aubry et al., 2016).

### 4.6 By what mechanism might 11β-HSD1 deficiency protect against the immediate and persistent effects of midlife chronic stress?

We have previously shown significantly lower increases in intra-hippocampal CORT levels in response to acute stress in 11β-HSD1 deficient mice compared to WT mice despite similar increases in plasma CORT levels (Yau et al., 2015b). This suggests that 11β-HSD1 deficient mice are more resilient to the detrimental effects of chronic stress/GCs on cognition and mood as the cumulative effects of increases in brain CORT levels will be avoided. It is noteworthy therefore that decreases in body weight gain that we and others have noted (Hoffman et al., 2014; McGuire et al., 2010; Zoladz et al., 2012; Zoladz et al., 2015) in response to stress in WT mice, are not seen in stressed 11β-HSD1 deficient mice which maintain body weight. This effect likely reflects altered GC action upon target tissues.

Several studies using mouse models of mood disorders have shown a causal role for GR in anxiety and depressive-like behaviors (Cattaneo and Riva, 2016). The reduced GR mRNA expression in the hippocampus of aged WT mice previously stressed in midlife could, in part, underlie the increased anxiety and depressive-like behaviors. Crucially, aged 11β-HSD1 deficient mice previously stressed in midlife resisted the decreased hippocampus GR mRNA expression and showed smaller stress-induced increases in plasma CORT, indicating an important role for local 11β-HSD1 generated GC in GR regulation.

### 4.7 Limitations of the study

The longitudinal nature of the study with repeated behavioral testing in the same animal from midlife and 6 months later in old age has both benefits and drawbacks. While it provides information on individual change in performance of multiple behaviors as a function of time following midlife stress exposure, the repeated testing may carry over effects complicating the interpretation of later testing (McIlwain et al., 2001). Spatial memory in the Y-maze is relatively stress free and mice can be re-tested (with new external cues around maze) without carry over effects. However, contextual fear conditioning has long lasting effects that could affect future shock-evoked fear conditioning so testing in midlife and 6 months later is a limitation that needs to be considered when analysing the data. It is well documented that stress does not produce global memory deficits but can enhance amygdala- and hippocampus-dependent contextual fear conditioning (Kim et al., 2015). Our finding of impaired spatial memory but enhanced fear memory in WT mice months after chronic stress support this notion. Other studies have also shown chronic stress induced spatial memory impairment (Conrad et al., 1996) and enhanced fear memory (Conrad et al., 1999) but in separate cohorts of animals. Another confounding factor to consider is the possibility of litter effects such as early life maternal care effects on behavior in later life (Liu et al., 2000) since the mice used in this study were not littermates. Although we cannot rule out litter effects, there is no evidence of this since non-stressed control mice (WT and KO) do not show significant differences in cognitive and affective measures.

### 4.8 Conclusion

11β-HSD1 deficiency protect against the long-lasting detrimental effects of midlife stress on cognitive function, anxiety and depressive-like behaviors into old age. Elucidating the mechanisms whereby 11β-HSD1 generated GCs in the brain contributes to the development of cognitive and affective dysfunction in aged mice many months after chronic stress in midlife may help in our understanding of anxiety-related disorders in the aged. It is plausible to consider 11β-HSD1 inhibitors in its treatment.

## Author’s contribution

NW, AH, CC, EA performed the experiments and analysed the data. JY, NW, CK, JRS contributed to study design and data interpretation. JY analysed the data and drafted the manuscript. JY, CK, JRS, AH, EA edited the manuscript. All authors have approved the final manuscript.

## Conflict of interest

None.

## Funding

This work was supported by a Biotechnology and Biological Sciences Research Council (BBSRC) Targeted Priority Studentship (BB/G016917/1) and the University of Edinburgh Centre for Cognitive Ageing and Cognitive Epidemiology, part of the cross council Lifelong Health and Wellbeing Initiative (G0700704). Funding from the BBSRC, Engineering and Physical Sciences Research Council (EPSRC), Economic and Social Research Council (ESRC), and Medical Research Council (MRC) is gratefully acknowledged. The funders had no role in the study design; in the collection, analysis and interpretation of data; in the writing of the report; and in the decision to submit the paper for publication.
