# Supplementary material for: Midlife stress alters memory and mood-related behaviors in old age: Role of locally activated glucocorticoids
Source: Psychoneuroendocrinology. 2018 Mar;89:13–22. doi: 10.1016/j.psyneuen.2017.12.018 (PMC5890827; doi:10.1016/j.psyneuen.2017.12.018)
Supplement: Supplementary file 1 [file mmc1.docx]

***Appendix A: Supplemental information***


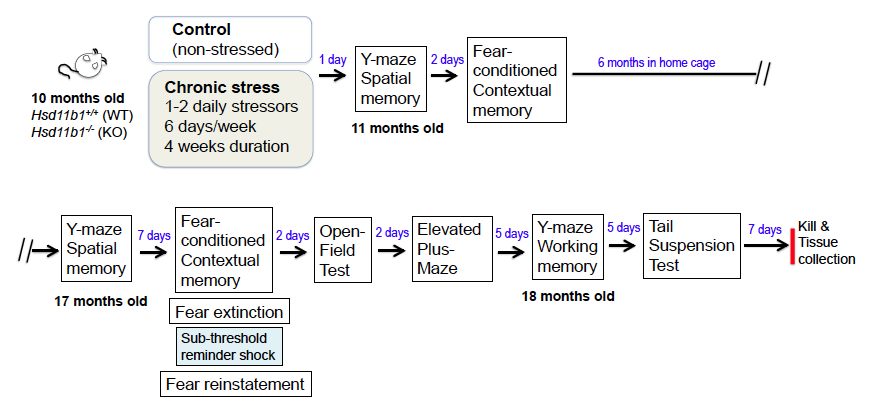


**Figure S1.** Experimental timeline. Wild-type (WT) and *Hsd11b1* knockout (KO) mice at 10 months old were exposed to a four-week period of unpredictable stress (n=10/genotype) or remained in their home cages as unhandled non-stressed controls (n=10/genotype). All behavioral testing was carried out between 7:00 am to 2:00 pm. Spatial and working memories were assessed in the Y-maze following chronic stress at the time points shown. Exposure of the mice to the elevated plus maze (EPM) and open field measured anxiety related behaviors while the tail suspension test (TST) measured depressive-like behaviours. Contextual fear conditioning where a neutral environment (context) is paired with an unconditioned aversive stimulus (electric footshock) was used to measure fear memory 4 days and 6 months after the cessation of stress. Fear memory extinction and reinstatement of fear post-extinction 24 h following a sub-threshold reminder footshock were assessed at 17 months old. All mice were left in their home cages between behavioural tests for the number of days shown.


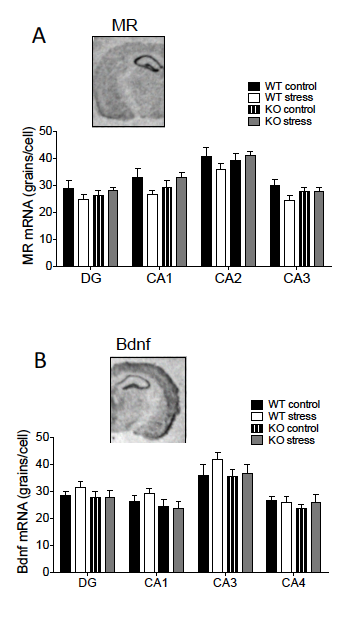


**Figure S2** Aged (18 months) wild-type (WT) and *Hsd11b1* knockout (KO) mice show no change in (A) MR and (B) *Bdnf* mRNA expression in the hippocampus 7 months after midlife chronic stress. Photos show autoradiographic representative regional distribution of MR and Bdnf mRNA expression in control brain sections. Hybridization signals (expressed as grains per cell) in photographic emulsion dipped slides were analysed under bright-field illumination. N=10/group. Data shown are mean ± SEM.
